# Coffee Silverskin Cellulose-Based Composite Film with Natural Pigments for Food Packaging: Physicochemical and Sensory Abilities

**DOI:** 10.3390/foods12152839

**Published:** 2023-07-26

**Authors:** Xinnan Liu, Hongbo Sun, Xiaojing Leng

**Affiliations:** 1 Key Laboratory of Functional Dairy, College of Food Science and Nutritional Engineering, China Agricultural University, Beijing 100083, China; xnliu0112@126.com (X.L.); b20183060491@cau.edu.cn (H.S.); 2 Key Laboratory of Precision Nutrition and Food Quality, Ministry of Education, China Agricultural University, Beijing 100083, China

**Keywords:** coffee silverskin cellulose, natural pigments, colored films, sensory properties, food packaging

## Abstract

To promote a circular economy, the use of agricultural by-products as food packaging material has steadily increased. However, designing food packaging films that meet consumers’ preferences and requirements is still a challenge. In this work, cellulose extracted from coffee silverskin (a by-product of coffee roasting) and chitosan were combined with different natural pigments (curcumin, phycocyanin, and lycopene) to generate a variety of composite films with different colors for food packaging. The physicochemical and sensory properties of the films were evaluated. The cellulose/chitosan film showed favorable mechanical properties and water sensitivity. Addition of natural pigments resulted in different film colors, and significantly affected the optical properties and improved the UV-barrier, swelling degree, and water vapor permeability (WVP), but there were also slight decreases in the mechanical properties. The various colored films can influence the perceived features and evoke different emotions from consumers, resulting in films receiving different attraction and liking scores. This work provides a comprehensive evaluation strategy for coffee silverskin cellulose-based composite films with incorporated pigments, and a new perspective on the consideration of the hedonic ratings of consumers regarding bio-based films when designing food packaging.

## 1. Introduction

Coffee consumption levels are extremely high around the world, and consequently, there is a large amount of agricultural residue generated from its production [1,2]. The reuse and recycling of coffee by-products to obtain high added-value products is thus of significant interest as it could help to achieve sustainable development and environmental protection goals [3,4]. Coffee silverskin (CS), a specific by-product of coffee, is the thin tegument of the coffee seeds, which is detached during the roasting process [5], and it usually contains cellulose (24%), hemicellulose (17%), and lignin (29%) [3]. During coffee production thousands of tons of silverskin are produced and could thus potentially be utilized as an abundant source of cellulose. However, the application of pure cellulose in food packaging has been impeded by its inherent limitations, which include poor mechanical performance and low water resistance [6,7]. Chitosan is an abundant natural polysaccharide that is synthesized by the deacetylation of chitin, which is mainly found in waste from the seafood industry [8,9,10]. The physicochemical and biological properties of chitosan have led to its increasing application in food packaging films [10,11,12,13]. Previous studies have revealed that the functional properties of some films, such as those that are polysaccharide (agarose and corn starch)- or protein (gelatin and whey protein isolate)-based, are enhanced when blended with chitosan [12,14,15,16,17,18]. As a basic function of food packaging, appropriate physicochemical properties of packaging films that help to protect products and prolong their shelf life are an essential requirement. The cellulose extracted from CS could thus be combined with chitosan to overcome these shortcomings and enhance film performance.

As packaging affects the visual appearance of products, it can impact the visual attractiveness and influence the perceptions and decisions of consumers [19,20]. In particular, the color of a product’s packaging is a critical driver of consumer attention and can affect specific responses to products [21,22,23]. The use of natural pigments that can endow packaging films with a wide range of colors and functions has become a focus of research due to food safety concerns and environmental damage associated with the use of synthetic dyes in food packaging [24,25]. Among these natural pigments, curcumin, an extract from turmeric, is a natural pigment that has a yellowish color [26], while lycopene is a tomato product with a red color [27], and phycocyanin (PC), which is found in cyanobacteria, rhodophyta, cryptophyta, and glaucophyte, has a blue color [28]. These active compounds have been widely used in films with desirable properties including antioxidant, antimicrobial, and color-changeable properties as smart packaging [29,30,31,32,33,34]. For instance, incorporation of curcumin into poly(lactic acid) or chitosan-based matrix materials shows excellent antioxidant and antimicrobial properties and has the potential to prolong the shelf life of products in the packaging [30,35]. Several studies have reported that films with lycopene had a protective effect against oxidation of foods with a high fat content [31,36]. Moreover, gelatin-based films that incorporated PC showed good antioxidant and antibacterial activity, showing promise as colored packaging [32]. While the physical and functional qualities of these colorful films are important, their perceived sensory characteristics and the emotions they evoke from consumers have frequently been ignored.

The check-all-that-apply (CATA) testing method has been widely used in consumer tests as it is considered easy for consumers to use. With this methodology consumers are asked to intuitively select terms including appropriate words or phrases, to describe a set of given samples [37]. The task can be easily completed by consumers without the need for advanced training and has been broadly used to obtain sensory profiles from consumers to evaluate foods such as cookies, yoghurt, pulse-based food products, cooked rice, and coffee [37,38,39,40,41]. In particular, the CATA method was also used to test the emotional responses of consumers towards the food packaging materials used for milk [42]. To identify the effective indicators that a consumer uses to form opinions on the visual appearance of food packaging, traditional self-reported measures have historically been used, while more recently eye-tracking techniques have also received increasing attention [43,44,45]. In particular, the eye-tracking technique has been widely used when assessing food packaging to record the eye movements of consumers’ natural viewing behaviors. Therefore, for designing novel packaging films, exploration of the sensory attitudes of consumers via diverse technologies can provide extremely valuable data.

The aim of this study was to investigate the physicochemical properties and consumer responses to a series of bio-based packaging films, obtained by combining cellulose extracted from CS with chitosan and natural pigments (curcumin, PC, and lycopene). Characterization of the multicolor composite films was conducted using scanning electron microscopy (SEM), X-ray diffraction (XRD), and Fourier transforms infrared (FT-IR) analyses. The influence of pigments on the color, mechanical properties, water sensitivity, hydrophobicity, and water vapor barrier were also explored. The relationships between the physicochemical and sensory characteristics were investigated via correlation and principal component analysis (PCA) analyses. Further to this, consumer perceptions (perceived features and evoked emotions) towards the films were obtained using the CATA test. Meanwhile, the liking assessment of the films and practical applications as a coffee powder packaging were measured and recorded using eye-tracking technology.

## 2. Materials and Methods

### 2.1. Materials

CS was gifted from Yunnan Miaichen Biotechnology Co., Ltd. (Yunnan, China). As a by-product from the factory, the obtained coffee products are a mixture with multiple coffee states (mainly Arabica). The NaOH, acetic acid, NaClO_2_, glycerol, and curcumin were purchased from Macklin (Shanghai, China). Chitosan with a molecular weight of 150 kD (deacetylation degree > 85%) was obtained from Jinan Haidebei Marine Bioengineering Co., Ltd. (Jinan, China). PC (purity [A620 nm/A280 nm] > 2.5) was obtained from Zhejiang Binmei Biotechnology (Taizhou, China). Lycopene (modified lycopene with water-solubility containing 10% lycopene with 40% OSA starch, and 10% maltodextrin (DE20) was purchased from Xi’an Xihai Bio-technique (Xi’an, China). All reagents were of analytical grade. Nestle brand instant coffee powder (60% Arabica coffee) and Mengniu Corp. brand milk powder (protein 32.6 g/100 g; fat 1.2 g/100 g; calcium 1100 mg/100 g) were acquired from a local supermarket.

### 2.2. Development of Composite Films with Different Natural Pigments

Cellulose was extracted from the CS in our laboratory. The process is shown in the supporting material, and was in line with a previous report with some modifications [46]. The obtained cellulose was dispersed in a beaker with de-ionized water and stirred for 2 days at a concentration of 2%. The cellulose solution was then homogenized using an FA25 high-speed disperser (Fluko Equipment Shanghai Co., Ltd., Shanghai, China) at 10,000 rpm for 5 min. The chitosan solution was prepared using 2% chitosan in an acetic acid solution (1%) with continuous stirring overnight to obtain a homogeneous solution. The cellulose solution was added into the chitosan solution to generate a film-forming solution, then glycerol was mixed into the solution at a concentration of 20% of the total solid mass. The solution was then cast into polyethylene rectangle Petri dishes (length of side of 13 cm) under an ambient environment of 25 ± 3 °C and 50% relative humidity (RH) for two days. The obtained films were designated as White film due to the white color of the film, which could be easily identified.

For colorful film formation, curcumin, phycocyanin, and lycopene were added into the chitosan solution with stirring, respectively. After the curcumin dissolved in the chitosan solution, only phycocyanin or both phycocyanin and lycopene were added into the obtained curcumin–chitosan solution to form a solution with a green or brown color. The colored chitosan solutions were mixed with cellulose to synthesize the colored films. The observed films presented different colors and were named the Yellow film, Blue film, Red film, Green film, and Brown film, accordingly. The components used to form the solutions of these films are presented in Table S1.

### 2.3. Characterization of the Films

#### 2.3.1. Surface and Cross-Section Morphology

The film surface and cross-section microstructures were acquired by Geminisem 500 (Carl Zeiss, Oberkochen, Germany) at an accelerating voltage of 15 kV. Prior to the test, a thin layer of gold was sprayed on small film pieces under vacuum condition.

#### 2.3.2. XRD and FTIR

The XRD patterns of the specimens were determined using by MiniFlex 600 X-ray diffractometer (Rigaku, Tokyo, Japan) using Cu Kα radiation (40 kV, 30 mA) with a scanning speed of 5°/min from 5 to 60°.

FTIR spectrometry was conducted by PerkinElmer Spectrum Two Infrared spectrometer (Perkin Elmer, Hopkinton, MA, USA) in a frequency range of 4000–600 cm^−1^ under an attenuated total reflection.

### 2.4. Thickness and Color Properties

Thickness was measured using a digital micrometer (0.001 mm accuracy). Five locations on each film were randomly evaluated to gain the average thickness.

The L*-value (lightness), a*-value (redness/greenness), and b*-value (yellowness/blueness) of the films were assessed using a portable meter (3nh Technology Corp., Shenzhen, China) with a standard white plate (L* = 94.08, a* = 0.35, b* = 2.18). The color difference (ΔE*) was calculated using [32]:

(1) $$\Delta E=\sqrt{{\Delta L}^{2}+{\Delta a}^{2}+{\Delta b}^{2}}$$

where ΔL, Δa, and Δb are the differences in the color parameters between the films and standard color plate, respectively.

The light transmittance of the films was recorded by a Varian Cary 50 UV/Vis spectrophotometer (Varian, CA, USA) with a spectra collection from 200 to 800 nm.

### 2.5. Mechanical Properties

Tensile and puncture tests were carried out using a texture analyzer (TA-XT PLUS/50, Brookfield Engineering Laboratories, Inc. Middleboro, MA, USA). To test tensile strength (TS) and elongation to break (EB), the films were cut into strips (1 × 5 cm), and the values were calculated using the following equations:

(2) $$\mathrm{TS}\;(\mathrm{MPa})=\frac{Fmax}{W\times T}$$

(3) $$\mathrm{EB}\;(\%)=\frac{L-L_{0}}{L_{0}}$$

where *Fmax* is the maximum force (N), *W* is the film width (mm), *T* is the film thickness (m), *L* is the final length (mm), and *L*_0_ is the initial length (mm).

Puncture tests were further completed to assess the mechanical properties of the films. The film samples (9 cm in diameter) were fastened in a holder with a circular opening. Films were then perpendicularly pierced by a puncture probe (5 mm). Puncture strength/thickness (N/mm) was obtained by dividing the strength by the film thickness to eliminate the effects resulting from thickness variations [47]. The puncture deformation (PD, %) was calculated using Equation (4) [48]:

(4) $$\mathrm{PD}=\frac{\sqrt{D^{2}+{L_{0}}^{2}}-L_{0}}{L_{0}}\times 100$$

where *D* is the distance of the probe until the film was pierced and *L*_0_ is the radius of the circular opening.

### 2.6. Water Content, Swelling Degree, and Water Solubility

The films (2 × 2 cm) were weighed (*M*1) and dried in an oven at 105 °C for 12 h. The dried samples were weighed *(M*2) and then immersed in water for 24 h at room temperature (25 ± 3 °C). The samples were weighed after their surfaces were cleaned with water (*M*3). These samples were then dried at 105 °C for 12 h (*M*4) and their values were calculated using Equations (5)–(7) [49]:

(5) $$\mathrm{Water}\;\mathrm{content}\;(\%)=\frac{M1-M2}{M1}\times 100$$

(6) $$\mathrm{Swelling}\;\mathrm{degree}\;(\%)=\frac{M3-M2}{M2}\times 100$$

(7) $$\mathrm{Water}\;\mathrm{solubility}\;(\%)=\frac{M2-M4}{M2}\times 100$$

where *M*1 is the initial weight of film (g), *M*2 is the dry weight of film (g), *M*3 is the water-soaked film weight (g), and *M*4 is the final dry weight of film (g).

### 2.7. Water Contact Angle and Water Vapor Permeability

Water contact angle (WCA) measurements for the films were evaluated using a contact-angle analyzer (Dataphysics OCA 25, Dataphysics, Filderstadt, German). The rectangular film samples (5 × 1 cm) were fixed on a platform, 3 μL droplets were gently dropped onto the film surface, and each film was analyzed in triplicate.

The water vapor permeability (WVP) was based on a previously documented method [50] with minor modifications. The samples were fixed in a permeation cup, added with anhydrous calcium chloride in the interior (0% RH). The permeation cups were placed in a desiccator with a saturated NaCl solution (75% RH) for 16 h, during which the cups were measured every 4 h for 12 h. Three replicates of each sample were examined using Equation (8) [50]:

(8) $$\mathrm{WVP}=\frac{W\times d}{A\times t\times \Delta p}$$

where *W* is the increased weight of the cup (g), *d* is the film thickness (m), *A* is the permeation area (m^2^), *t* is the time (s), and Δ*P* is the difference in vapor pressure between the two sides of the film at 2339.27 Pa (20 °C).

### 2.8. Sensory Evaluation

#### 2.8.1. Participants

There were a total of 77 participants (44% male and 56% female, aged 19–21 years old and SD = 0.7), all of whom were undergraduates at China Agricultural University majoring food science. This study was reviewed and approved by the Medical Ethical Committee of China Agricultural University (Project identification code: 20220204).

#### 2.8.2. Preparation of Samples

The colorful films were prepared according to the process described in Section 2.2. Pictures of each of the six films were displayed on a screen for the eye-tracking test. The samples were cut into rectangles (1 × 4 cm) and placed on a plate with random three-digit numbers.

#### 2.8.3. Procedure

The participants were introduced to the raw materials and the preparation processing of the films so that they were informed about the material. Eye movement and mouse click data were collected using an EyeSo Ec-80 eye-tracking device (Brain Craft Technology Co., Ltd., Beijing, China) with Eyeso Studio software (version 3.3, Eyeso Technology, Beijing, China). Participants were asked to sit 60 cm from the eye tracking monitor (21″ full HD screen, 1920 × 1080-pixel resolution) and avoid moving during the test. Before the task, participants were asked to finish the 9-point calibration procedure. First, the instructions presented on the screen were as follows: “Look at the pictures and click on your favorite”. Pictures of the colorful films or coffee powder packaging were then presented on the screen (Figure S1). Then, the participants were instructed to smell, touch, and stretch until breaking the real rectangle film samples, which were presented in the plate as described in 2.8.2. The participants were asked to select and click the intensity of factors using a nine-point intensity scale on the screen (the points on the scale are described in detail in Table S2). These factors included the appearance (perceived lightness, PL), flavor (odor acceptance, OA), touch (roughness of air surface, RAS; roughness of plate surface, RPS), stretch (perceived tensile strength, PTS), and overall liking (OL) of the films. The instructions given to the participants were as follows: “Please click on the intensity that best describes the film presented to you”. After one film was rated, participants were asked to complete the first CATA question containing 12 terms relating to the sensory description of the film (smooth, easy to tear, deep color, novel, firm, cheap, environmental, fragile, fragrant, light color, foul, rough, advanced, and dry) and a second CATA question with 14 emotional attributes (comfortable, disappointed, fresh, displeased, appealing, disliked, satisfied, warm, relaxing, delightful, surprised, and disgusted) and participants were asked to select the words that best describe their responses to the film. The CATA tests were conducted with the following instructions: “Please click all the attributes that you consider to describe the films given to you”. The words in the CATA tests were presented on a screen in a randomized arrangement. Before each task, a fixation cross was presented in the center of the screen for 2000 ms to fixate the gaze of participants. The whole test was completed within 15–20 min. After completing the survey online, the participants were then provided with a questionnaire to request detailed background information such as their age, gender, height, and weight.

For the purpose of this work, the index of the eye-movements for the total duration time and fixation count were used as the eye-tracking data [51,52]. A set of areas of interest (AOI) was defined to measure participants’ gazes on each film in isolation and when used to pack coffee powder. The fixation duration is the amount of time participants fixed their eyes on AOI [53]. Fixation count measures the number of participants who fixated within an AOI [54].

### 2.9. Statistical Analysis

Statistical analysis was performed using a one-way analysis of variance (ANOVA) and Tukey’s post hoc test to identify the significant differences among samples (*p* < 0.05). CATA binary data were analyzed using Cochran’s Q test following Marascuilo’s procedure to compare the pairwise samples [55]. Data from the CATA tests were based on the selection frequency and were analyzed using correspondence analysis (CA). The parameters of gazing behavior (total duration time and fixation count) were analyzed using the Friedman non-parametric test. The one-way ANOVA, Cochran’s Q, CA, and Friedman non-parametric tests were conducted using SPSS Software v. 20 (IBM Corp., Chicago, IL, USA). Physicochemical data and sensory intensity data were analyzed using correlation analysis and PCA with Origin 2021 (OriginLab Corp., Northampton, MA, USA).

## 3. Results and Discussion

### 3.1. Characterization of the Composite Films

#### 3.1.1. Film Microstructures

**Figure 1:**
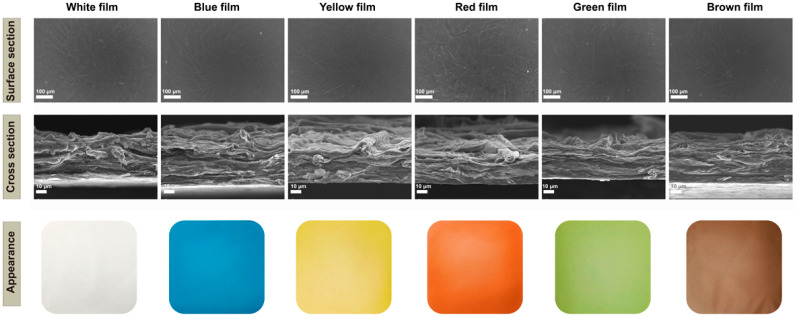
Scanning electron microscopy micrographs (surface appearance and cross sections) and visual appearance of the cellulose/chitosan film and cellulose/chitosan film with natural pigments.

The surface and cross-section views of the original White film showed the penetration of chitosan between the cellulose and the formation of a dense fibrillar network (Figure 1). The natural pigments were found to be uniformly dispersed in the matrix, which resulted in the film surfaces being much rougher (Figure 1). In particular, the addition of lycopene resulted in the surface of the film having more particles and the presence of agglomerates, which may be due to the concentration used. The cross-section of the colorful films showed a layered structure and no significant changes compared with the White film after absorption of the pigments, which was consistent with a previous report [56].

#### 3.1.2. XRD Analysis

**Figure 2:**
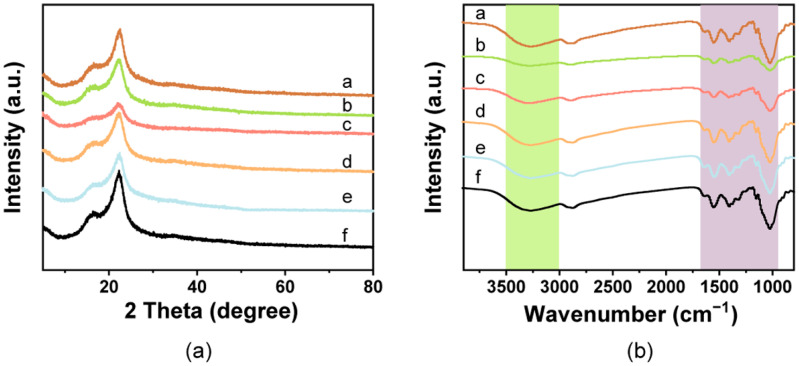
Structural characterization of the composite films. (**a**) FTIR spectra and (**b**) XRD patterns observed from the cellulose/chitosan film and cellulose/chitosan film with natural pigments. a: White film, b: Blue film, c: Yellow film, d: Red film, e: Green film, f: Brown film.

The XRD diffraction patterns showed that the spectra of the pure film had characteristic absorption peaks for cellulose near 16.8° and 22.4°, which corresponded to (110) and (200) planes of typical cellulose I (Figure 2a) [46]. No obvious diffraction peaks for chitosan were found in the spectra, which may be due to its existence in an amorphous state, as previously reported [57,58]. The peak intensities of the films decreased after addition of the pigments, which may be due to the reduction of cellulose crystallinity. However, there were no sharp diffraction peaks in the films containing pigments.

#### 3.1.3. FTIR Analysis

The spectral bands of all films showed peaks at 3400–3450 cm^−1^ for the -OH stretching of cellulose and chitosan and the abundance of hydrogen bonds in the film, indicating interfacial interactions and compatibility between the cellulose and chitosan [57,59,60] (Figure 2b). The observed peak at 2800–2900 cm^−1^ represented the stretching vibration of C-H [12]. The peaks at 1650, 1560, and 1400 cm^−1^ corresponded to amide I, amide II, and amide III, respectively, which belong to the chitosan [61,62]. The peaks observed at 1130 and 1023 cm^−1^ were due to the presence of C-O-C ring stretching (β-glycosidic linkage vibration) and C-O stretching, respectively [63,64]. Except for slight variations in the peak intensities, no noteworthy new peaks were found in the colorful films containing the natural pigments, which confirmed that there was no obvious change in the chemical structure. The results also suggested that there is a physical interaction between the cellulose/chitosan matrix and natural pigments.

### 3.2. Thickness and Optical Properties

The thickness of the films ranged from 24.5 to 31.9 μm (Table 1). With the addition of the pigments, the thickness tended to decrease (expect for the Yellow film), with the Blue and Red films showing significant decreases (*p* < 0.05) when compared with the White film. A similar effect was observed in a previous report, where the addition of free lycopene led to a decrease in the thickness of cassava starch films [31]. The change in the Blue film was in contrast to the previous results for bovine gelatin film [32].

Color has previously been found to greatly affect consumer preference for food packaging materials [65]. The films had homogeneous visual appearances and smooth surfaces with the incorporation of the natural pigments into the film solution, which significantly changed the L*, a*, and b* values (*p* < 0.05) (Figure 1; Table 1). The films with added curcumin and lycopene showed significant increases in a* and b* (*p* < 0.05). The PC led to decreases in L*, a*, and b*, where negative a* values indicate the green region and negative b* values indicate the blue region, and a lower L*indicates a dark color. The film had a green color with the addition of curcumin and phycocyanin. Brownish films were obtained with lower L* values and higher a* and b* values, which changed due to the mixtures of the three pigments. Depending on the addition of the natural pigments, all films showed significant increases in ΔE* (*p* < 0.05). values. The Blue film had the highest ΔE* value.

The light transmittance spectra of the films are shown in Figure S2. All films exhibited low transmittance values < 12% in the UV region (UV-A, 315–400 n; UV-B, 280–315 nm; and UV-C, 200–280 nm) [66]. In comparison with the White film, the transparency was decreased in the colored films. Subsequently, the UV barrier properties of these films were increased. The UV barrier properties and lower transparencies of the films could block ultraviolet light to protect the quality of the packaged food.

### 3.3. Mechanical Properties of the Films

**Figure 3:**
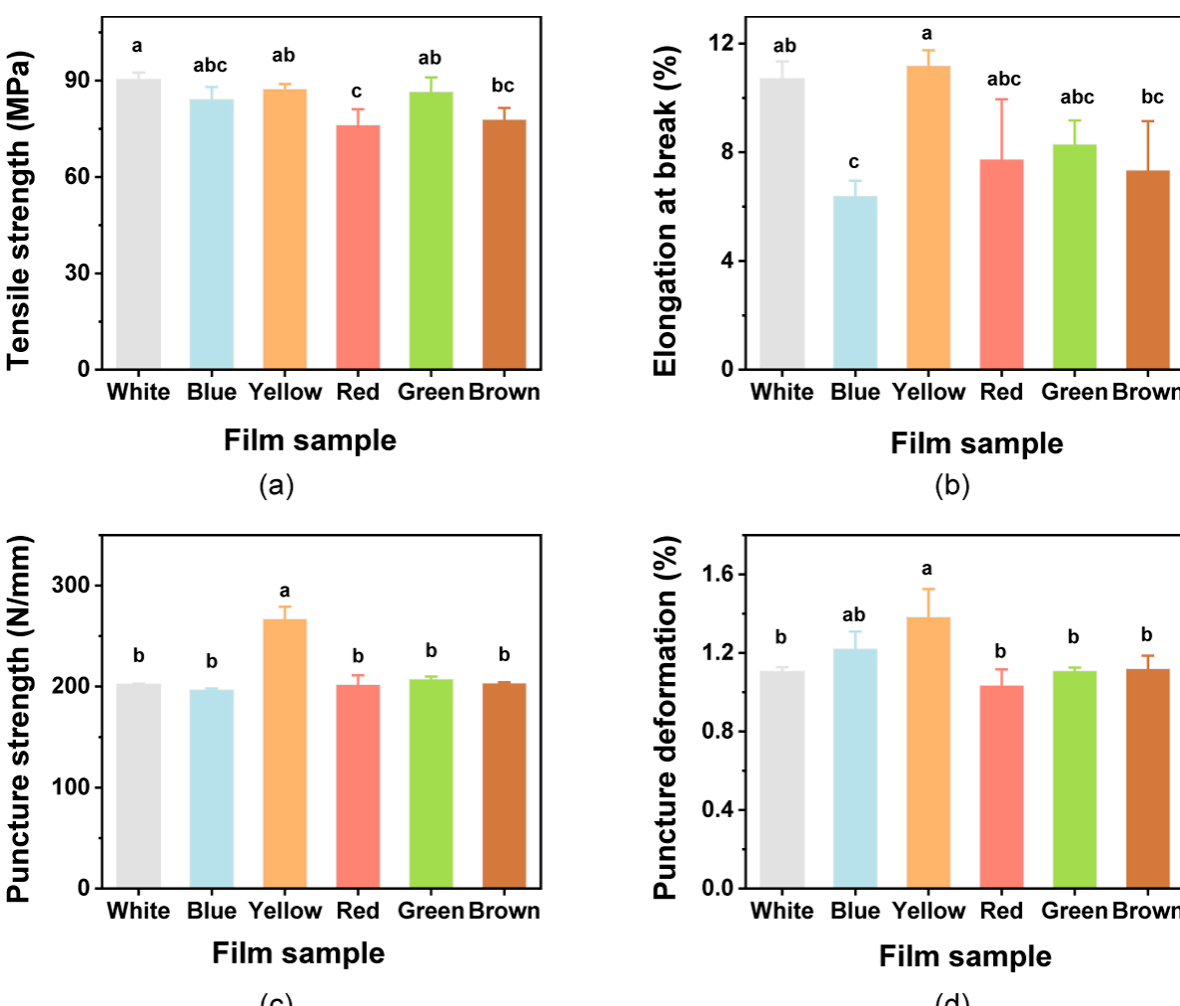
Mechanical properties of the composite films. (**a**) Tensile strength; (**b**) Elongation at break; (**c**) puncture strength; and (**d**) puncture deformation. Different lowercase letters above bars indicate significant differences (*p* < 0.05).

Films used for packaging should possess mechanical strength and extensibility to ensure that they can protect foods and meet application needs [67]. The mechanical properties of the colorful films were thus evaluated. The TS values of the films showed no differences when compared with the White film (*p* > 0.05), except for Red and Brown films (Figure 3a). When compared with the EB of the White film, significant differences were only found with the Blue film (Figure 3b). Addition of the natural pigments to the films caused some slight decreases in TS and EB values as previous reports [31,35]. This may be attributed to the decrease in interchain spacing and interchain interactions [32,68]. The results obtained for PS and PD are shown in Figure 3c,d. There were no significant impacts on the PS and PD of the films with the pigments when compared with the White film (*p* > 0.05), except for the rise in the Yellow film. However, the value of EB was also increased due to the effects of the added curcumin, indicating that the curcumin could improve the flexibility of the films.

### 3.4. Water Content, Swelling Degree, and Water Solubility

The water content (WC) of the films, which is related to their water resistance ability, plays a key role in the shelf-life stability of coated and packaged materials [69]. The WC of the films ranged from 12.52 to 15.99%, and those with curcumin had the highest moisture content (Table 2). The incorporation of pigments into the films resulted in an increase in WC, except with lycopene, but the differences were not significant when compared to the White film.

The White film had the highest swelling degree (SD) values, and showed significant differences with the colored films (*p* < 0.05; Table 2). The films with pigments showed decreased swelling, which may be due to the increased crosslink density that occurs with the addition of the pigments [70,71].

Films used in food packaging should be insoluble in water; in other words, they should not dissolve in wet environments or upon contact with aqueous-based foods, and solubility will ideally be as low as possible. The solubility of all films tested ranged from 10.89 to 13.18%, and there was no significant difference between the White film and those with pigments (Table 2). The results indicate that all films could potentially be widely utilized as food packaging due to their low levels of water solubility.

### 3.5. WCA and WVP

**Figure 4:**
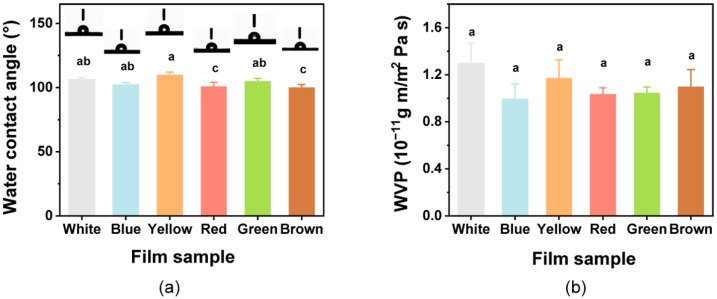
(**a**) Water contact angle and (**b**) water vapor permeability (WVP) for the composite films. Different lowercase letters above bars indicate significant differences (*p* < 0.05).

To assess the hydrophobic/hydrophilic characteristics of the film, the WCA of films was assessed (Figure 4a). The WCA values for all films were >90°, indicating that the colorful films all had hydrophobic surfaces. The films with added natural pigments showed significant differences (*p* < 0.05) in WCA. The WCA of the film increased slightly, with the addition of curcumin. Similar behavior to that observed with the curcumin addition has previously been found in various other polymer films, such as poly(lactic acid) and cellulose-based films [35,72].

The functional role of a packaging film is to preserve the product from humidity to avoid moisture transferring between the environment and the food [69]; thus, the WVP of all films was investigated (Figure 4b). The WVP of films ranged from 0.99 × 10^−11^ g m/m^2^ Pa s to 1.30 × 10^−11^ g m/m^2^ Pa s. The White film had the highest WVP value, but ultimately it was not significantly (*p* > 0.05) influenced by the introduction of the pigments, and these results are in accordance with those of a previous study [73]. The films in this study could potentially be used in food packaging and food preservation to extend the shelf life.

### 3.6. Sensory Properties of the Multicolor Films

#### 3.6.1. Correlation between the Sensory Attributes and Physical Properties

**Figure 5:**
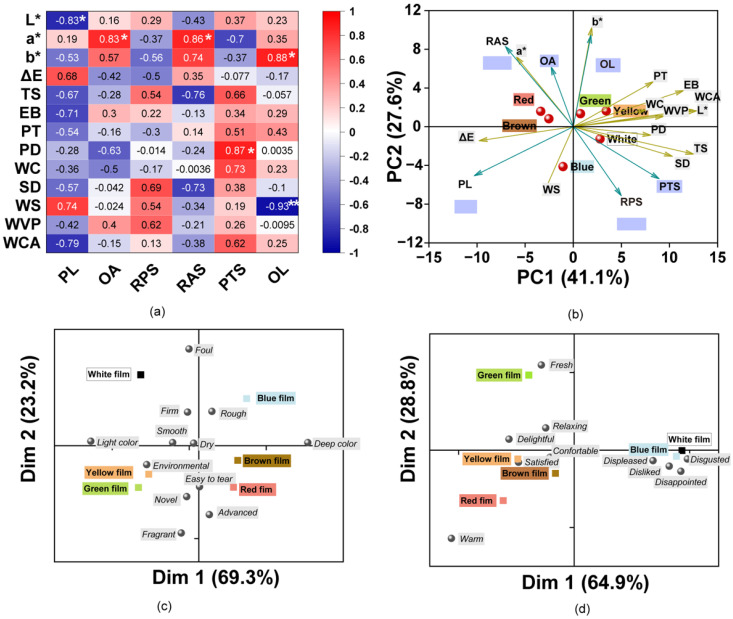
(**a**) Matrix of Pearson correlation coefficients for the physicochemical and sensory parameters. The color of the squares indicates the intensity of their correlation. (* indicates *p* < 0.05; ** indicates *p* < 0.01); (**b**) Biplot of the first two dimensions (F1 and F2) based on principal component analysis of the film samples and properties. Arrows show characteristics for divergence between composite films with different colors. The farther the characteristics are from the origin, the more essential for the differentiation of the film. PL: perceived lightness, OA: odor acceptation, RBS: roughness of air surface, RPS: roughness of plate surface, PTS: perceived tensile strength, OL: overall liking. (**c**,**d**) Biplot representation of the participants’ perceptions to films with descriptions for the first two dimensions (Dim 1 and Dim 2) of the correspondence analysis. (**c**) Features of the films; (**d**) emotions evoked by the films.

The correlations between the multivariate data for the sensory and physicochemical properties were explored (Figure 5a). The color values L*, a*, b*, and ΔE were evidently correlated with the perceived color, and consequently, L* was significantly negatively correlated with PL with a coefficient of 0.83 (*p* < 0.05). Likewise, a* was significantly positively correlated with OA and RAS for the film (*p* < 0.05). OL showed a significant positive correlation with b*, which indicated that participants preferred the bright-colored films in this study. The color was also related with a tough physical feeling and participant preference. In addition, mechanical properties (TS, EB, PT, and PD) were positively correlated with PTS, especially PD, which was found to be significantly positively correlated with PTS. SD, WS, and WVP were positively correlated with the films plate surface and showed opposing correlations with that of the air surface of the film. Likewise, WC, SD, WS, WVP, and WCA were positively correlated with PTS. The results indicate that the water sensitivity of the films affected the feel of the film surface and perceived tensility. These results indicate that the sensory attitudes were correlated with the physicochemical parameters. In addition, the color of the films was also found to influence the perceived sense of the participants, which indicated cross-modal effects of color and touch [74].

#### 3.6.2. PCA Analysis

As shown in Figure 5b, the variance contribution of PC1 and PC2 accounted for approximately 68.7% of the total data variance. The colored films were separated into four different groups. Therein, the White film was separated from the others. One group corresponds to Red and Brown films, which had a higher a*, OA, and smooth surface (RAS). The Green and Yellow films were found to belong to the other group and had higher values for b*, L*, mechanical properties (PT and EB), WC, WVP, WCA, and OL. However, the Blue film showed an opposing trend with higher ΔE, PL, and WS. The ΔE were in accordance with the higher PL according to the participants. The results indicate that the incorporation of pigments contributed to the differences in the physical and sensory characteristics.

#### 3.6.3. Analysis of the Features and Emotions Evoked by the Films

To further explore the sensory perceptions of consumers to these films, attitudes in response to the film features and the emotions evoked were assessed using CATA tests. The results from the Cochran’s Q test revealed that there were 12 features that were significant attributes of the films with different colors (*p* < 0.05). A bi-plot of CA shows the differences and similarities between the films based on the frequency of selection of the CATA terms by mouse click (Figure 5c). The first two dimensions explained 93.5% of the total variance. Dimension 1 (Dim 1) mainly divided the films into two groups, those with a deep color or a light color. The results were similar for the tendency of ΔE * values, as the Green and Yellow films were found to be closer, while the Brown and Red films were separated together. Dimension 2 (Dim 2) was positively associated with the foul, firm, and rough characteristics. Except for the White and Blue films, the other film colors had negative values. The low liking scores for the blue films may be associated with an undesirable smell and dark color. The Yellow and Green films were more associated with novel and environmental. The Red and Brown films were described as easier to tear, which was related to the results of the synergistic effects of TS and EB. The results of consumer perceptions of films were majorly influenced by the different pigments.

According to previous studies, the various colors can evoke different emotions (positive or negative) in consumers, and thus the color of food packaging can affect the acceptance of food products [23,75,76]. Consequently, the emotions of the consumer in response to these colorful films were explored using the CATA test. The results showed that 10 out of the 12 terms were perceived and identified by the panelists as being significantly different (*p* < 0.05) between all samples. According to the perceived emotional differences, the films were divided into three groups using CA (Figure 5d). The CA explained 93.7% of the variability with 69.4% on Dim 1 and 28.8% on Dim 2. The White and Blue films were near descriptions such as displeased, disgusted, disappointed, and disliked, which could thus explain the lower liking for these films, and the selection of these words may be due to their foul and rough properties. The Green film was described as fresh and relaxing which may explain why it was liked the most. The Yellow, Red, and Brown films were characterized as being delightful, satisfied, comfortable, and warm. The Yellow film was associated with the terms delightful and satisfied, which was in accordance with the previous report which mentioned that the color yellow is associated with words such as joy, happy, and joyful [23,77]. The various colors can be classified into warm and cool. For example, colors in the red, orange, and yellow ranges are warm, while those in the white, green, and blue ranges are cool [23,78]. The results were corroborated as the Red film was linked to warm, while the White, Blue, and Green films were distant from the Red film and located in other quadrants.

#### 3.6.4. Liking Assessment

**Figure 6:**
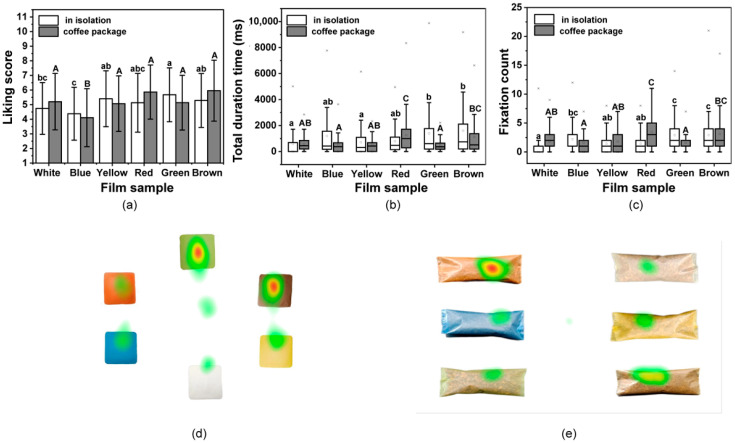
Liking of the films determined was evaluated using the mouse click and eye-tracking data. (**a**) Mean liking scores for films in isolation and when used to pack coffee based on click data. (**b**) Boxplots showing the total duration time and (**c**) fixation counts for the participants gaze on pictures of films in isolation and when used to pack coffee. Different lowercase letters above bars indicate significant differences between films (*p* < 0.05); Different uppercase letters above bars indicate significant differences between films used in packed coffee (*p* < 0.05). Heatmaps for (**d**) films in isolation and (**e**) when films are used to pack coffee. Red areas indicate the highest gaze values, while green areas indicate low gaze values.

To assess the effects of these colorful films in relation to consumer performance, participants were asked how much they liked the films. As shown in Figure 6a, for the single film, the liking values varied from 4.38 to 5.68, and the Green film obtained the highest score, while the Blue film had the lowest. There were significant differences in the hedonic ratings among all films (*p* < 0.05). After being used to package coffee, the liking values were found to range from 4.11 to 5.96 (Figure 6a). The Brown film when used to pack the coffee received the highest liking score, while the Blue film still had the lowest score. The obtained liking values of films may be dominated by the perceived features and evoked emotion from consumers to films.

To explore the participants liking for the composite films, the gazing behavior of participants when exposed to pictures of the films and packed coffee were measured using eye-tracking technique. The results are presented using boxplots in Figure 6b,c. The values for the Green and Brown films were higher, and the results were similar for the liking values. The White film received the least amount of attention from participants, when in isolation or used for coffee packaging. The Brown and Red coffee packaging received more attention and showed the same trend as seen with the liking values. Heat maps were generated using the data from all participants to visually identify their favorite films and coffee packages (Figure 6d,e). Thus, there were long gaze values for the Green and Brown films when viewed in isolation, while the Brown and Red received longer gaze values when used for coffee packaging, which indicates the same tendency with liking choice. The choice of packaging color might be attributed to the category of product itself for the cognitive associations of consumers [79]. Films were found to generally be more attractive to the consumer if their color was similar to that of the packaged product [80]. Moreover, these results indicate that the assessments for the colored films in isolation and when used practically as packaging, obtained different liking values from the consumer than that of white film, which supports a hedonic consideration of consumers when using bio-based films in food packaging.

Furthermore, as illustrated in Figure S3a, the prepared films can be fabricated into a diverse range of shapes for coffee powder. Furthermore, the milk powder can also be encapsulated into films (Figure S3b), which highlights the various application potentials of coffee silverskin cellulose-based films in the food packaging industry.

## 4. Conclusions

The CS cellulose-based films incorporated with natural pigments were successfully prepared. The introduction of pigments endowed various color to films with various physicochemical and sensory properties. Addition of pigments led to an increase in UV-barrier and decrease in SD of films. Meanwhile, these films still maintained good hydrophobicity, low WVP, and acceptable mechanical properties when compared with cellulose/chitosan film. Moreover, the colored films received higher liking scores, abundant sensory perceptions, and more attractions from consumers. Overall, in this study, we comprehensively considered both the physicochemical parameters and sensory attitudes of participants in regard to CS cellulose-based films providing a new and practical design strategy for bio-based packaging for practical application.

## Figures and Tables

**Table 1:** Film thickness and color values (L*, a*, b*, ΔE) of composite films.

Samples	Thickness (μm)	L*	a*	b*	ΔE
White film	31.00 ± 1.49 b	90.05 ± 0.25 f	−0.52 ± 0.09 c	3.51 ± 0.52 b	4.35 ± 0.37 a
Blue film	24.50 ± 0.79 a	64.83 ± 1.67 b	−21.42 ± 0.49 a	−26.89 ± 0.94 a	46.64 ± 1.84 e
Yellow film	31.19 ± 0.31 b	86.57 ± 0.57 e	0.51 ± 0.36 d	29.67 ± 2.24 d	28.49 ± 2.31 c
Red film	25.25 ± 2.75 a	67.14 ± 1.35 c	22.61 ± 1.06	19.32 ± 0.92 c	38.92 ± 1.93 d
Green film	26.56 ± 3.42 ab	79.66 ± 0.54 d	−8.90 ± 0.16 b	18.78 ± 0.69 c	23.85 ± 0.86 b
Brown film	28.94 ± 2.25 ab	60.51 ± 0.63 a	15.16 ± 0.16 e	19.16 ± 0.42 c	40.43 ± 0.66 d

The data are written as mean ± the standard deviation (n = 5 for thickness, and n = 3 for L*, a*, b*,and ΔE). Different lowercase letters within a column indicate significant differences (*p* < 0.05).

**Table 2:** Water content, swelling degree, and water solubility of composite films.

Samples	Water Content (%)	Swelling Degree (%)	Water Solubility (%)
White film	13.35 ± 2.20 a	43.27 ± 3.44 a	12.85 ± 0.3 a
Blue film	14.03 ± 3.10 a	35.57 ± 2.43 bc	13.18 ± 2.0 a
Yellow film	15.99 ± 1.98 a	35.76 ± 3.07 c	11.90 ± 0.5 a
Red film	13.04 ± 1.45 a	30.86 ± 2.32 ab	12.69 ± 1.0 a
Green film	13.71 ± 2.29 a	38.86 ± 3.42 bc	10.89 ± 1.4 a
Brown film	13.94 ± 1.80 a	32.83 ± 0.66 bc	12.14 ± 0.8 a

The data are shown as the mean ± the standard deviation (n = 3). Different lowercase letters within a column indicate significant differences (*p* < 0.05).

## Data Availability

All related data and methods are presented in this paper. Additional inquiries should be addressed to the corresponding author.

## Supplementary Materials

The following supporting information can be downloaded at: https://www.mdpi.com/article/10.3390/foods12152839/s1, Figure S1 Illustration of eye-tracking experiment. (a) Task for selecting favorite film; (b) task for selecting the favorite coffee package. Figure S2 Light transmittance of the films. Figure S3 Pictures of films with practical applications. (a) Films used to pack coffee powder; (b) films used to pack milk powder. Table S1 Components of natural pigments in film forming solutions. Table S2 Descriptions of rating scale points.
