# Supplementary material for: Coffee Silverskin Cellulose-Based Composite Film with Natural Pigments for Food Packaging: Physicochemical and Sensory Abilities
Source: Foods. 2023 Jul 26;12(15):2839. doi: 10.3390/foods12152839 (PMC10417091; doi:10.3390/foods12152839)
Supplement: Supplementary file 1 [file foods-12-02839-s001.zip › foods-2498095-supplementary.pdf]

Supplementary Materials

Coffee Silverskin Cellulose-Based Composite Film with Natural Pigments for Food Packaging: Physicochemical and Sensory Abilities

Xinnan Liu [1,2], Hongbo Sun [1,2] and Xiaojing Leng [1,2,*]

[1] Key Laboratory of Functional Dairy, College of Food Science and Nutritional Engineering, China Agricultural University, Beijing 100083, China; xnliu0112@126.com (X.L.); b20183060491@cau.edu.cn (H.S.)
[2] Key Laboratory of Precision Nutrition and Food Quality, Ministry of Education, China Agricultural University, Beijing 100083, China
* Correspondence: lengxiaojingcau@163.com; Tel.: +86-10-6273-7761

Isolation of cellulose form coffee sliverskin

The process was followed previous report with some modifications [1]. Before treatment, the dried coffee silverskin was milled using mixer-grinder (400Y, Yongkang Platinum Ou Hardware Products Co., LTD, China) and sieved using 60 mesh to obtain coffee silverskin powder. The powder was boiled in 60 °C water for 1 h. Then, the obtained mass was stirred three times with NaOH solution (4 wt%) for 2 h at 80 °C. The residue was collected and bleached treated with a mixed solution 4 times for 2 h at 80 °C to gain white colored cellulose. The solution with two equal parts, one acetate buffer contained 27 g NaOH and 75 mL glacial acetic acid, then diluted to 1 L of distilled water, another is 1.7 wt% $NaClO_2$ solution. Finally, the cellulose was washed with deionized water until the pH of the filtrate to neutral. After freeze drying, the cellulose was stored at room temperature in a dry condition.

Table S1. Components of natural pigments in film forming solutions

Sample	Composition (mg/mL)		
	Phycocyanin	Curcumin	Lycopene
White film	-	-	-
Blue film	1.5	-	-
Yellow film	-	0.2	-
Red film	-	-	8
Green film	0.25	0.2	-
Brown film	0.5	0.2	4

Table S2. Descriptions of rating scale points.

Rated task	Score		
	1	5	9
Perceived lightness	Extremely light	Moderate	Extremely dark
Odor acceptance	Extremely rejective	Moderate	Extremely acceptable
Roughness of air surface of film	Extremely smooth	Moderate	Extremely rough
Roughness of plate surface of film	Extremely smooth	Moderate	Extremely rough
Perceived tensile strength	Extremely fragile	Moderate	Extremely firm
Overall liking	Dislike Extremely	Not dislike not like	Like Extremely

Figure S1. Illustration of eye-tracking experiment. (**a**) Task for selecting favorite film; (**b**) task for selecting favorite coffee package.

Figure S2. Light transmittance of the films.

Figure S3. Pictures of films with practical applications. (**a**) Films used to pack coffee powder; (**b**) films used to pack milk powder.

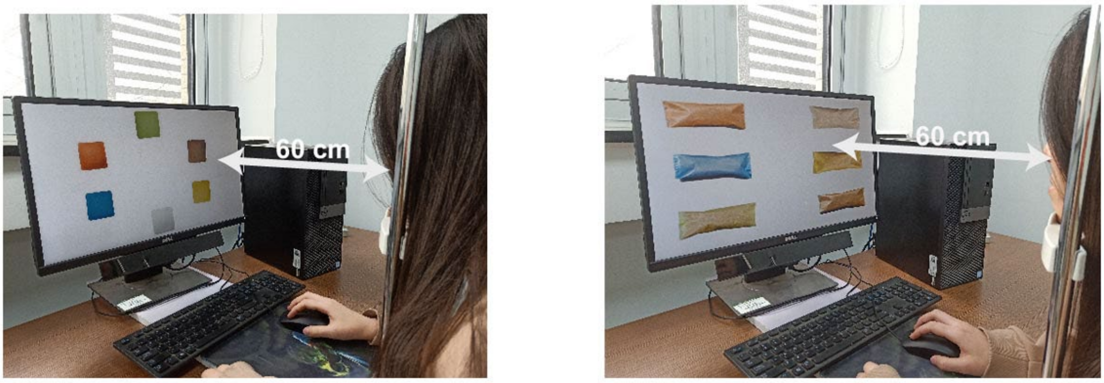

(a) (b)

Figure S1.

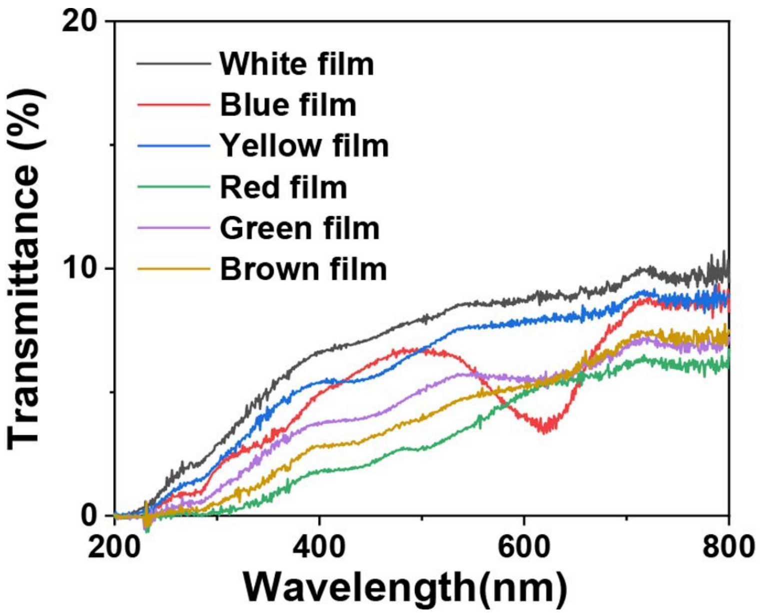
20
10
0
200
400
600
800
Transmittance (%)
Wavelength(nm)
White film
Blue film
Yellow film
Red film
Green film
Brown film

Figure S2.

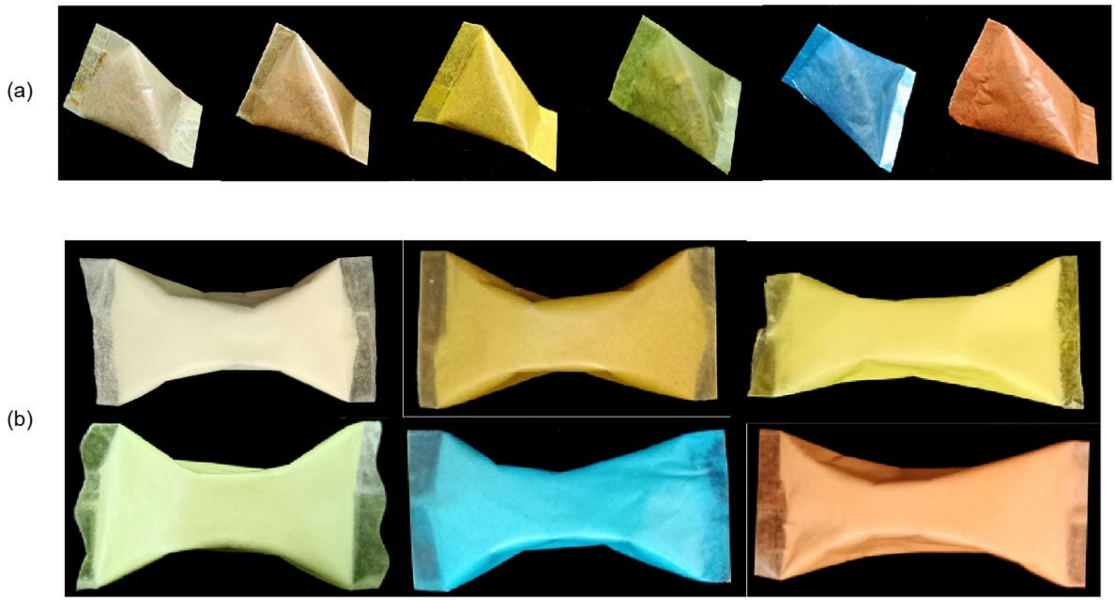

Figure S3.

Reference

1. El Achaby, M.; Ruesgas-Ramon, M.; Fayoud, N.-E.H.; Figueroa-Espinoza, M.C.; Trabadelo, V.; Draoui, K.; Ben Youcef, H. Bio-sourced porous cellulose microfibrils from coffee pulp for wastewater treatment. *Cellulose* **2019**, *26*, 3873-3889, doi:10.1007/s10570-019-02344-w.
